# Maternal Inflammation Exaggerates Offspring Susceptibility to Cerebral Ischemia–Reperfusion Injury via the COX-2/PGD2/DP_2_ Pathway Activation

**DOI:** 10.1155/2022/1571705

**Published:** 2022-04-09

**Authors:** Yuke Li, Wen Luo, Jiahua Zhang, Ying Luo, Wenli Han, Hong Wang, Hui Xia, Zhihao Chen, Yang Yang, Qi Chen, Huan Li, Lu Yang, Congli Hu, Haifeng Huang, Zhe Peng, Xiaodan Tan, Miaomiao Li, Junqing Yang

**Affiliations:** ^1^Chongqing Key Laboratory of Biochemistry and Molecular Pharmacology, College of Pharmacy, Chongqing Medical University, Chongqing 400016, China; ^2^Department of Clinical Pharmacy, The Third Hospital of Mianyang/Sichuan Mental Health Center, Mianyang, Sichuan 621000, China; ^3^Laboratory Animal Center, Chongqing Medical University, Chongqing, 400016, China; ^4^Pharmacy Department of Guizhou Provincial People's Hospital, Guiyang, Guizhou 550000, China

## Abstract

The pathogenesis of cerebral ischemia–reperfusion (I/R) injury is complex and does not exhibit an effective strategy. Maternal inflammation represents one of the most important factors involved in the etiology of brain injury in newborns. We aimed to investigate the effect of maternal inflammation on offspring susceptibility to cerebral I/R injury and the mechanisms by which it exerts its effects. Pregnant SD rats were intraperitoneally injected with LPS (300 *μ*g/kg/day) at gestational days 11, 14, and 18. Pups were subjected to MCAO/R on postnatal day 60. Primary neurons were obtained from postnatal day 0 SD rats and subjected to OGD/R. Neurological deficits, brain injury, neuronal viability, neuronal damage, and neuronal apoptosis were assessed. Oxidative stress and inflammation were evaluated, and the expression levels of COX-2/PGD2/DP pathway-related proteins and apoptotic proteins were detected. Maternal LPS exposure significantly increased the levels of oxidative stress and inflammation, significantly activated the COX-2/PGD2/DP_2_ pathway, and increased proapoptotic protein expression. However, maternal LPS exposure significantly decreased the antiapoptotic protein expression, which subsequently increased neurological deficits and cerebral I/R injury in offspring rats. The corresponding results were observed in primary neurons. Moreover, these effects of maternal LPS exposure were reversed by a COX-2 inhibitor and DP_1_ agonist but exacerbated by a DP_2_ agonist. In conclusion, maternal inflammatory exposure may increase offspring susceptibility to cerebral I/R injury. Moreover, the underlying mechanism might be related to the activation of the COX-2/PGD2/DP_2_ pathway. These findings provide a theoretical foundation for the development of therapeutic drugs for cerebral I/R injury.

## 1. Introduction

Stroke represents the second leading cause of morbidity, mortality, and health care costs worldwide [1]. Stroke is usually referred to as a condition caused by the occlusion or hemorrhage of vessels supplying the brain with blood [2]. Ischemic stroke accounts for approximately 85% of all strokes [3]. Thrombolytic therapy is one of the most effective treatments for acute ischemic stroke, and it can promote cell survival by restoring the blood supply to the ischemic area [4, 5]. However, the restoration of blood flow and oxygenation results in an exacerbation of tissue injury and a profound inflammatory response (reperfusion injury) [6]. Therefore, elucidating the molecular mechanism of cerebral ischemia–reperfusion (I/R) injury and the development of a novel drug target are of great importance [7, 8].

During the early period of brain development, abnormally elevated levels of proinflammatory cytokines likely derail neurodevelopment, leading to brain dysfunction or latent susceptibility to inflammation-triggered brain injury later in life [9] and even resulting in higher susceptibility to brain diseases, such as schizophrenia. For example, fetal cortical neurons exposed to high concentrations of interleukin- (IL-) 1*β*, IL-6, and tumor necrosis factor-*α* (TNF-*α*) experience disrupted dendritic development [10]. Longitudinal studies in rat and mouse prenatal immune models showed that prenatal inflammation-induced behavioral and pharmacological abnormalities were progressive and only shown during adolescence and adulthood in offspring [11–14]. Maternal infection and inflammation can cause neurological disorders and abnormal brain development through a disrupted fetal microenvironment. Maternal inflammatory exposure, an altered microenvironment, and subsequent neuronal injury have been considered to be the pathophysiologic basis for neurodevelopmental disorders [15–18]. Studies have shown that exacerbated maternal inflammatory responses confer susceptibility to preeclampsia [19]. However, it is not clear whether maternal inflammatory exposure can influence offspring susceptibility to cerebral I/R injury. If yes, what are the mechanisms involved?

The pathophysiology of cerebral I/R injury is very complex and involves multiple mechanisms, including bursts of reactive oxygen species, calcium overload, excitotoxicity, and neuroinflammation [20–22]. Oxidative stress and inflammatory reactions stimulate further release of inflammatory factors in cerebral I/R injury [23–25]. Moreover, suppression of oxidative stress and excessive inflammation is generally effective at alleviating cerebral I/R injury [26–28]. Cyclooxygenase-2 (COX-2) upregulation is a hallmark of inflammation, and the inhibition of COX-2 might be effective in inhibiting COX-2-dependent prostanoids induced by inflammatory stimuli [29–31]. Studies indicate that the expression of COX precedes the appearance of inflammatory factors in neurons and glial cells after brain injury and secretes inflammatory factors to cause an inflammatory cascade [32]. Therefore, activation of COX may be closely related to cerebral I/R injury. Prostaglandin D2 (PGD2), one of the COX-2-mediated metabolites of arachidonic acid, exerts physiological effects through the D-type prostanoid receptor (DP_1_ and DP_2_) [33–35]. There is accumulating evidence that inflammatory response cascades are stimulated, leading to further apoptosis and necrosis of cells [36]. Inflammation and apoptosis are involved in the mechanisms of cerebral I/R injury [7, 37–39].

In the present study, we first established a “double-hit” model, which consisted of maternal inflammation induced by lipopolysaccharide (LPS) and cerebral I/R injury in offspring. Second, we observed the effect of maternal LPS exposure on susceptibility to cerebral I/R injury in offspring rats. Next, we observed the effect of maternal LPS exposure on susceptibility to cerebral I/R injury in primary neurons of offspring and further administered COX-2 inhibitor, DP_1_ agonist/antagonist, and DP_2_ agonist/antagonist separately to preliminarily assess the mechanisms of increased susceptibility from the COX-2/PGD2/DP pathway in primary neurons of offspring.

## 2. Materials and Methods

### 2.1. Animals

A total of 30 pregnant SD rats were provided by the Laboratory Animal Centre of Chongqing Medical University (SCXK (Chongqing) 2018-0003). Pups were generated by SD pregnant rats. All rats were housed in separate cages under a 12-h light/dark cycle at 22 ± 2°C and 50 ± 10% humidity with food and water ad libitum. This study was approved by the Ethics Committee of Chongqing Medical University, and all animal handling and treatment procedures were in accordance with the Health's Guide for the Care and Use of Laboratory Animals.

### 2.2. Animal Model of Maternal Intraperitoneal Administration of LPS

The model was established as previously described [40–42]. The rats were injected with LPS (Escherichia coli, serotype 055: B5; Sigma–Aldrich, St Louis, MO, USA) (300 *μ*g/kg, intraperitoneally) at gestational Days 11, 14 and 18.

### 2.3. Middle Cerebral Artery Occlusion and Reperfusion (MCAO/R) Model

Offspring adult male rats were subjected to MCAO/R on postnatal day 60 (220-250 g, *n* = 24). The model was established as previously described [43]. The right common carotid artery of rats was inserted with a 2 cm nylon filament until it obstructed the middle cerebral artery. After 90 min, the filament was withdrawn and reperfused for 24 h.

### 2.4. Rat Primary Neuron Culture

Primary neuron culture was performed as described previously with slight modifications [44]. Primary neurons were obtained from postnatal day 0 SD rats. The hippocampus and cortices were isolated and immersed in PSB buffer (Beijing Dingguo Biotechnology Co., Ltd., China). Cells were cultured in neurobasal medium (Gibco, USA) supplemented with 2% B-27 supplement (Gibco, USA) at 37°C. The small tissue mass and dead cells were removed by changing the culture medium 4-6 h after seeding. The neuron cultures were used for subsequent experiments to identify the purity of neurons after 7 d.

### 2.5. Oxygen-Glucose Deprivation and Reoxygenation (OGD/R) Model

An OGD/R model was established in rat primary neurons. The medium was replaced with glucose-free DMEM (Gibco, Gaithersburg, MD, USA). Subsequently, the primary neurons were transferred to an anaerobic incubator equilibrated (1% O_2_, 94% N_2_, and 5% CO_2_) for 2 h at 37°C. After 2 h, the medium was replaced with neurobasal medium supplemented with 2% B-27 and returned to a normoxic incubator (95% O_2_ and 5% CO_2_) for 24 h at 37°C.

### 2.6. Experimental Grouping and Treatment

*Experiment* 1 is as follows: To observe the effect of maternal LPS exposure on the susceptibility to cerebral I/R injury in offspring rats, pups were randomized into 4 groups. The N + sham group is as follows: maternal intraperitoneal saline injections and pups subjected to sham operation. The LPS + sham group is as follows: maternal intraperitoneal LPS injections and pups subjected to sham operation. The N + MCAO/R group is as follows: maternal intraperitoneal saline injections and pups subjected to MCAO/R. The LPS + MCAO/R group is as follows: maternal intraperitoneal LPS injections and pups subjected to MCAO/R, *n* = 12 in each group.

Experiment 2 is as follows: to observe the effect of celecoxib on the susceptibility to OGD/R in primary neurons of offspring treated with prenatal LPS, the primary neurons of offspring were randomized into 7 groups. The N + N group is as follows: maternal intraperitoneal saline injections and offspring primary neuron cultures. The LPS + N group is as follows: maternal intraperitoneal LPS injections and offspring primary neuron cultures. The N + OGD/R group is as follows: maternal intraperitoneal saline injections and OGD/R-treated offspring primary neurons. LPS + OGD/R is as follows: maternal intraperitoneal LPS injections and OGD/R-treated offspring primary neurons. The LPS + N + celecoxib group is as follows: maternal intraperitoneal LPS injections and offspring primary neuron cultures administered celecoxib. The N + OGD/R + celecoxib group is as follows: maternal intraperitoneal saline injections and OGD/R/celecoxib-treated offspring primary neurons. The LPS + OGD/R + celecoxib group is as follows: maternal intraperitoneal LPS injections and OGD/R/celecoxib-treated offspring primary neurons.

Experiment 3 is as follows: to observe the effect of DP_1_ agonist/antagonist or DP_2_ agonist/antagonist on the susceptibility to OGD/R in primary neurons of offspring treated with prenatal LPS, the offspring primary neurons were randomized into 6 groups. The LPS + N + BW245C/BWA868C/DK-PGD2/AZD1981 group is as follows: maternal intraperitoneal LPS injections and offspring primary neurons administered DP_1_ agonists, DP_1_ antagonists, DP_2_ agonists, and DP_2_ antagonists, respectively. The N + OGD/R + BW245C/BWA868C/DK-PGD2/AZD1981 group is as follows: maternal intraperitoneal saline injections and OGD/R-treated offspring primary neurons administered DP_1_ agonists, DP_1_ antagonists, DP_2_ agonists, and DP_2_ antagonists, respectively. The LPS + OGD/R + BW245C/BWA868C/DK-PGD2/AZD1981 group is as follows: maternal intraperitoneal LPS injections and OGD/R-treated offspring primary neurons administered DP_1_ agonists, DP_1_ antagonists, DP_2_ agonists, and DP_2_ antagonists, respectively. The N + N group, LPS + N group, N + OGD/R group, and LPS + OGD/R group were treated in the same manner as in experiment 2.

### 2.7. Neurological Deficit Assessment

A modified neurological grading score was used to evaluate the neurological test, *n* = 12 in each group: 0: no deficit and normal spontaneous movements, 1: left front leg was flexed but not circling clockwise, 2: left front leg was flexed and spontaneously circling clockwise, 3: spin clockwise longitudinally, and 4: unconsciousness and no spontaneous movement.

### 2.8. Cerebral Infarction Volume Assessment

Triphenyltetrazolium chloride (TTC) staining, as previously described [45], was used to evaluate cerebral infarction volume, *n* = 3 in each group. The brains were rapidly extracted and cut into five 2 mm coronal slices and then placed in 2% TTC solution (Sigma–Aldrich, St. Louis, MO, USA) for 20 min at 37°C and fixed with 4% paraformaldehyde. Cerebral infarction volume was analyzed by ImageJ software (NIH, Baltimore, MD, USA).

### 2.9. Histopathologic Examination

Hematoxylin and eosin (HE) staining, as previously described [46], was used to evaluate histopathologic damage in the cortex and hippocampus, *n* = 3 in each group. Rats were anesthetized with 5% sodium pentobarbital and transcardially perfused with 250 mL of PBS (0.1 M; pH 7.4) containing heparin followed by 4% paraformaldehyde. After being sacrificed, the brains of the rats were isolated and blocked in 4% paraformaldehyde. After paraffin embedding was performed, brain sections (5 *μ*m) were stained with HE. Normal cells showed intact and clear nuclear membranes and nucleoli, and damaged cells showed nuclear pyknosis, cellular vacuolization, and disordered arrangement. The neuronal damage rate was calculated as the number of damaged cells divided by the total number multiplied by 100.

### 2.10. Neuronal Viability Measurement

A Cell Counting Kit-8 (CCK-8, Sigma–Aldrich, USA) assay was performed to determine cell viability. Ten microliters of CCK-8 solution was added to each well, which was then incubated at 37°C for 60 min without light. The optical density (OD) values of each well were measured at 450 nm by using an enzyme-linked immunoassay reader (BioTek, USA).

### 2.11. Neuronal Damage Measurement

Lactate dehydrogenase (LDH) leakage rate (Beyotime, China) assays were performed to determine neuronal damage and follow the operation steps of the LDH test kit instructions. The optical density (OD) values of each well were measured at 490 nm by using an enzyme-linked immunoassay reader (BioTek, USA).

### 2.12. Neuronal Apoptosis Measurement

Flow cytometry analysis was performed to determine neuronal apoptosis and followed the operation steps of the FITC-Annexin V Apoptosis Detection Kit (BD Pharmingen, San Diego, CA, USA).

TUNEL staining was performed by following the operation steps of the TUNEL fluorescence FITC kit (Roche, Indianapolis, IN) to determine neuronal apoptosis. The nuclei of primary neurons were stained with DAPI (Sigma–Aldrich). TUNEL-positive cells were observed and captured with a fluorescence microscope (Nikon, Inc., Japan).

### 2.13. Oxidative Stress Measurement

The oxidative stress level was evaluated by the measurement of malondialdehyde (MDA) content and superoxide dismutase (SOD) activity and was conducted according to the operation steps of relevant detection kits (Jiancheng Bioengineering Ltd., Nanjing, China).

### 2.14. Inflammation Measurement

Inflammation levels in brain tissue homogenates, cell supernatants, and culture medium were evaluated by the measurement of PGD2, TNF-*α*, IL-1*β*, and IL-6 following the operation steps of ELISA kits (Jiangsu Mei Biao Biological Technology Co., Ltd.)

### 2.15. Protein Expression Measurement

Total protein samples were extracted from the cortex and hippocampus using RIPA lysis buffer. Protein concentrations were detected by using a BCA Protein Assay Kit (Beyotime, China). Proteins were separated and transferred onto a polyvinylidene fluoride (PVDF) membrane (Millipore, USA). After blocking with 5% bovine serum albumin (BSA), the membrane was incubated overnight at 4°C with the primary antibody. Subsequently, the HRP-conjugated secondary antibodies (1: 1000, Proteintech, China) were incubated with the membranes for 1 h at room temperature. Finally, the levels of target proteins were visualized with a gel imaging apparatus (Bio–Rad, USA). After three washes with TBST, the membranes were incubated with HRP-conjugated secondary antibodies (1: 1000, Proteintech, China) for 1 hour at RT and were then washed with TBST an additional 3 times. Finally, the expression of proteins was visualized with a gel imaging apparatus (Bio–Rad, USA). The primary antibodies used were as follows: antibody against COX-2 (1: 1000, Abcam, UK), antibody against DP_1_ (1: 1000, Abcam, UK), antibody against DP_2_ (1: 1000, Santa, UK), antibody against caspase-3 (1: 1000, Abcam, UK), antibody against cleaved caspase-3 (1: 1000, Cell Signaling Technology, UK), antibody against caspase-9 (1: 1000, Santa, USA), antibody against Bcl-2 (1: 2000, Abcam, UK), and *β*-actin (1: 3000, Proteintech, USA).

### 2.16. Statistical Analysis

All data are expressed as the mean ± SD. GraphPad Prism 6 (GraphPad Software, USA) was used for statistical analysis. One-way analysis of variance (ANOVA) followed by Tukey's test was used to compare multiple groups, and a *t-*test was used to compare two groups. Data with nonnormal distributions were analyzed by a nonparametric test. *p* < 0.05 indicated a significant difference.

## 3. Results

### 3.1. Maternal LPS Exposure Increased Neurological Deficit and Cerebral Infarction Volume in MCAO/R Offspring Rats

**Figure 1:**
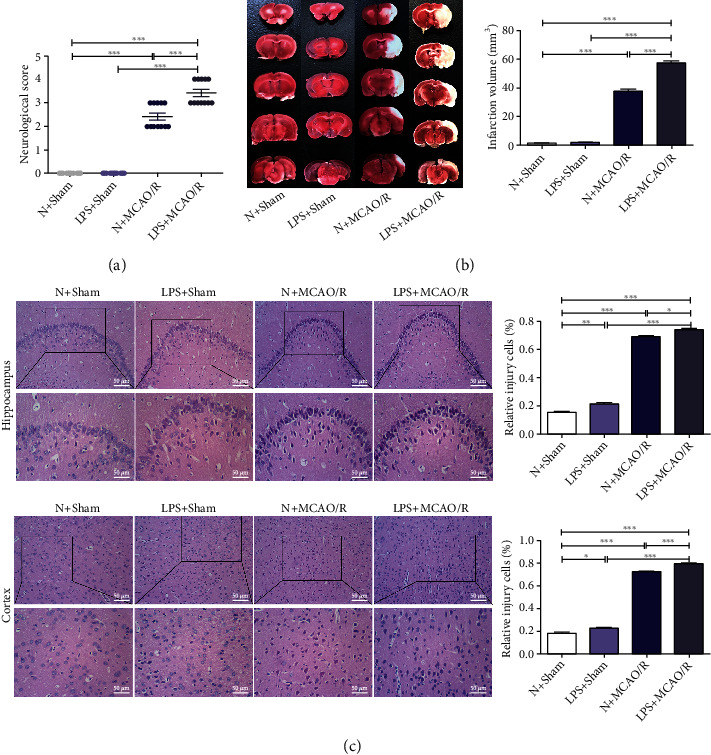
Effect of maternal LPS exposure on neurological deficit scores, cerebral infarction volume, and histopathologic damage in MCAO/R offspring rats. (a) Maternal LPS exposure increased neurological deficits, *n* = 12. (b) Maternal LPS exposure increased cerebral infarction volume, *n* = 3. (c) Maternal LPS exposure increased histopathologic damage in the hippocampus and cortex, *n* = 3. Scale bar = 50 *μ*m and 10 *μ*m (×200 and ×400). Data are expressed as the mean ± SD, ^∗^*p* < 0.05, ^∗∗^*p* < 0.01, ^∗∗∗^*p* < 0.001.

As shown in Figures 1(a) and 1(b), the neurologic deficit scores and cerebral infarction volume were not significantly different between the LPS + sham group and the N + sham group (*p* > 0.05). The neurological deficit scores (*p* < 0.001) and cerebral infarction volume (*p* < 0.001) were significantly increased in the N + MCAO/R group and the LPS + MCAO/R group compared with the N + sham group and the LPS + sham group. The neurological deficit scores and cerebral infarction volume were significantly increased in the LPS + MCAO/R group compared with the N + MCAO/R group (*p* < 0.001).

### 3.2. Maternal LPS Exposure Increased Histopathologic Damage in MCAO/R Offspring Rats

The histopathologic damage in the cortex and hippocampus is presented in Figure 1(c). The cells showed significant nuclear pyknosis, vacuolization, and disordered arrangement in the LPS + sham group compared with the N + sham group (*p* < 0.05 and *p* < 0.01). The cells showed significant nuclear pyknosis, vacuolization, and disordered arrangement in the N + MCAO/R group and LPS + MCAO/R group compared with the N + sham group and the LPS + sham group (*p* < 0.001). The cells showed significant damage in the LPS + MCAO/R group compared with the N + MCAO/R group (*p* < 0.05 and *p* < 0.001).

### 3.3. Maternal LPS Exposure Increased Oxidative Stress and Neuroinflammation in MCAO/R Offspring Rats

**Figure 2:**
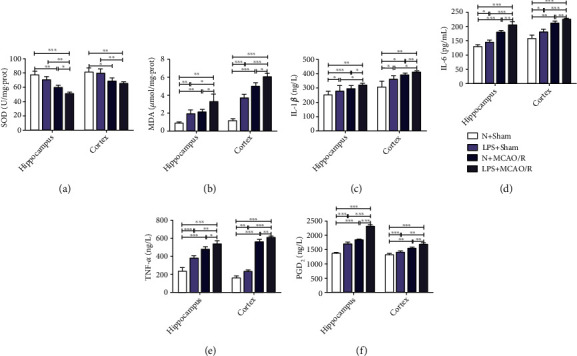
Effect of maternal LPS exposure on oxidative stress and inflammation in the hippocampus and cortex of MCAO/R offspring rats. (a, b) Maternal LPS exposure decreased SOD activity but increased MDA content, *n* = 6. (c)–(f) Maternal LPS exposure increased the levels of IL-1*β*, IL-6, TNF-*α*, and PGD2, *n* = 6. Data are expressed as the mean ± SD, ^∗^*p* < 0.05, ^∗∗^*p* < 0.01, ^∗∗∗^*p* < 0.001.

The results in the cortex and hippocampus are presented in Figures 2(a)–2(f). The activity of SOD was not significantly different (*p* > 0.05), but the levels of MDA (*p* < 0.01 and *p* < 0.001) and PGD2, TNF-*α*, IL-1*β*, and IL-6 (*p* < 0.05, *p* < 0.01, and *p* < 0.001) were significantly increased in rats in the LPS + sham group compared with those of rats in the N + sham group. SOD activity (*p* < 0.05 and *p* < 0.01) significantly decreased, but levels of MDA (*p* < 0.05, *p* < 0.01 and *p* < 0.001) and levels of PGD2, TNF-*α*, IL-1*β*, and IL-6 (*p* < 0.05, *p* < 0.01, and *p* < 0.001) were significantly increased in rats in the N + MCAO/R group and the LPS + MCAO/R group compared with those of rats in the N + sham group and the LPS + sham group. The activity of SOD (*p* < 0.05) was significantly decreased, but the levels of MDA (*p* < 0.05) and PGD2, TNF-*α*, IL-1*β*, and IL-6 (*p* < 0.05, *p* < 0.01, and *p* < 0.001) were significantly increased in rats in the LPS + MCAO/R group compared with those of rats in the N + MCAO/R group.

### 3.4. Maternal LPS Exposure Increased the Expression of COX-2, DP_1_, DP_2_, Caspase-3, Cleaved Caspase-3, Caspase-9, and Bcl-2 in MCAO/R Offspring Rats

**Figure 3:**
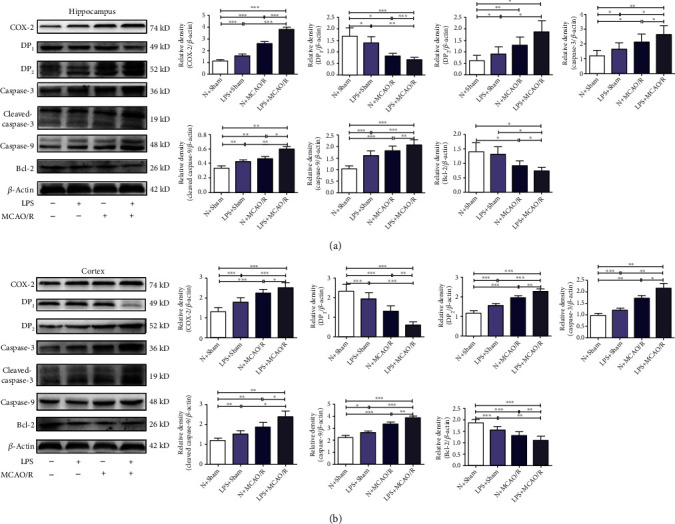
Effect of maternal LPS exposure on the expression of COX-2, DP_1_, DP_2_, caspase-3, cleaved caspase-3, caspase-9, and Bcl-2 in the hippocampus and cortex of MCAO/R offspring rats. (a) Maternal LPS exposure increased the expression of COX-2, DP_2_, caspase-3, cleaved caspase-3, and caspase-9 but decreased DP_1_ and Bcl-2 in the hippocampus. (b) Maternal LPS exposure increased the expression of COX-2, DP_2_, caspase-3, cleaved caspase-3, and caspase-9 but decreased DP_1_ and Bcl-2 in the cortex. Data are expressed as the mean ± SD, *n* = 4. ^∗^*p* < 0.05, ^∗∗^*p* < 0.01, ^∗∗∗^*p* < 0.001.

The results in the cortex and hippocampus are presented in Figures 3(a) and 3(b). The expression of COX-2, DP_2_, caspase-3, cleaved caspase-3, and caspase-9 was significantly increased, but the expression of DP_1_ and Bcl-2 was significantly decreased in rats in the LPS + sham group compared with that of rats in the N + sham group (*p* < 0.05, *p* < 0.01, and *p* < 0.001). The expression of COX-2, DP_2_, caspase-3, cleaved caspase-3, and caspase-9 was significantly increased, but the expression of DP_1_ and Bcl-2 was significantly decreased in rats in the N + MCAO/R group and the LPS + MCAO/R group compared with that of rats in the N + sham group and the LPS + sham group (*p* < 0.05, *p* < 0.01, and *p* < 0.001). The expression of COX-2, DP_2_, caspase-3, cleaved caspase-3, and caspase-9 was significantly increased, but the expression of DP_1_ and Bcl-2 was significantly decreased in the LPS + MCAO/R group compared with the N + MCAO/R group (*p* < 0.05, *p* < 0.01, and *p* < 0.001).

### 3.5. Celecoxib Increased Neuronal Viability and Decreased Neuronal Damage and Neuronal Apoptosis in OGD/R Offspring Primary Neurons Treated with Prenatal LPS

**Figure 4:**
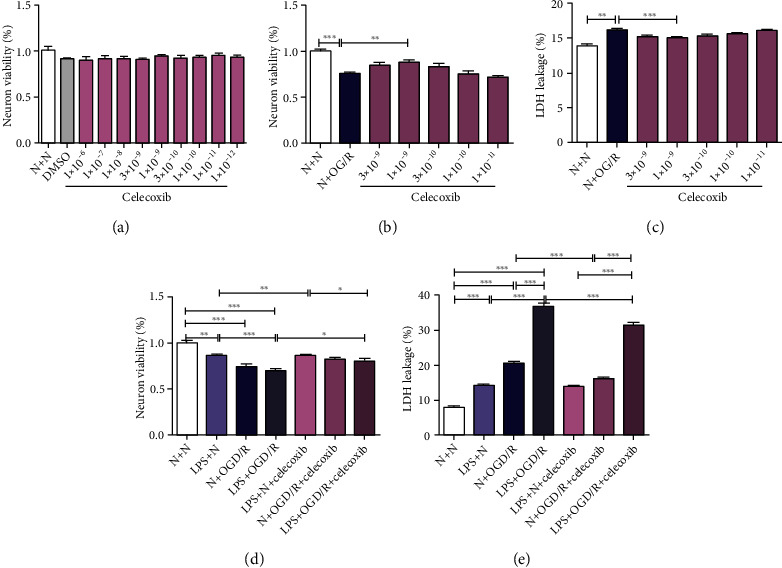
Effect of celecoxib on neuronal viability and neuronal damage in OGD/R offspring primary neurons treated with prenatal LPS. (a) No toxicity was observed at a celecoxib concentration of less than 1 × 10^−9^ M. (b, c) Celecoxib (concentrations less than 1 nM) increased neuronal viability but decreased neuronal damage in OGD/R-treated primary neurons. (d, e) Celecoxib increased neuronal viability but decreased neuronal damage in OGD/R primary neurons treated with prenatal LPS injection. Data are expressed as the mean ± SD, *n* = 10. ^∗^*p* < 0.05, ^∗∗^*p* < 0.01, ^∗∗∗^*p* < 0.001.

Primary neuron morphological characteristics were observed after 1, 3, 5, and 7 days of culture, and purity, identified by NeuN immunofluorescence staining after 7 days of culture, was greater than 95% (Figure S1A-B). To determine the most efficient treatment time using OGD/R, the neuronal viability and neuronal damage of primary neurons subjected to 2 or 3 h of OGD followed by 24 h of reoxygenation were evaluated. The neuronal viability was significantly decreased (*p* < 0.001), but neuronal damage was significantly increased in the N + OGD/R (2 h) group and the N + OGD/R (3 h) group (*p* < 0.001) compared with the N + N group (Figure S2A-B). Moreover, neuronal viability was lower in the N + OGD/R (3 h) group than in the N + OGD/R (2 h) group (Figure S2A-B). Primary neurons subjected to OGD/R for 2 h were chosen for the follow-up experiments. No cell toxicity was observed at celecoxib concentrations ranging from 1 × 10^−12^ M to 1 × 10^−6^ M (*p* > 0.05) (Figure 4(a)). Celecoxib at a concentration of 1 × 10^−9^ M significantly increased neuronal viability (*p* < 0.01) but significantly decreased neuronal damage in OGD/R-treated primary neurons (*p* < 0.001) (Figures 4(b) and 4(c)). Celecoxib (1 × 10^−9^ M) was chosen for the follow-up experiments.

**Figure 5:**
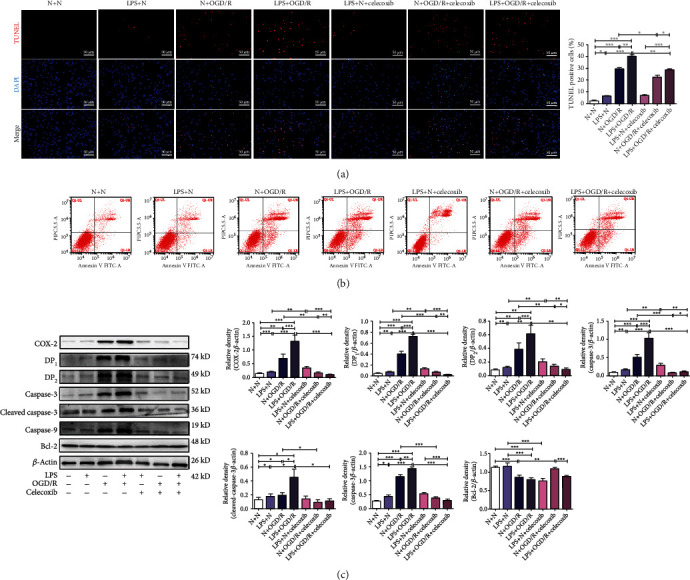
Effect of celecoxib on neuronal apoptosis and the COX-2 pathway in OGD/R offspring primary neurons treated with prenatal LPS. (a) Celecoxib inhibited neuronal apoptosis detected by TUNEL staining, *n* = 3. Scale bar = 50 *μ*m. (b) Celecoxib inhibited neuronal apoptosis detected by flow cytometry. (c) Expression of COX-2, DP_1_, DP_2_, caspase-3, cleaved caspase-3, caspase-9, and Bcl-2 detected by western blotting, *n* = 4. Data are expressed as the mean ± SD. ^∗^*p* < 0.05, ^∗∗^*p* < 0.01, ^∗∗∗^*p* < 0.001.

As shown in Figures 4(d), 4(e), and 5(a), the neuronal viability (*p* < 0.01) was significantly decreased, but neuronal damage (*p* < 0.001) and the number of TUNEL-positive cells (*p* < 0.05) were significantly increased in the LPS + N group compared with the N + N group. The neuronal viability (*p* < 0.001) was significantly decreased, but neuronal damage (*p* < 0.001) and the number of TUNEL-positive cells (*p* < 0.001) were significantly increased in rats in the N + OGD/R group and rats in LPS + OGD/R group compared with that of rats in the N + N group and the LPS + N group. The neuronal damage (*p* < 0.001) and the number of TUNEL-positive cells (*p* < 0.01) were significantly increased in rats in the LPS + OGD/R group compared with that of rats in the N + OGD/R group.

The neuronal viability (*p* < 0.01) was significantly increased, but neuronal damage (*p* > 0.05) and the number of TUNEL-positive cells (*p* > 0.05) had no statistical difference in the LPS + N + celecoxib group compared with the LPS + N group. The neuronal viability (*p* > 0.05) had no statistical difference, but neuronal damage (*p* < 0.001) and the number of TUNEL-positive cells (*p* < 0.05) were significantly decreased in the N + OGD/R + celecoxib group compared with the N + OGD/R group. The neuronal viability was significantly increased (*p* < 0.05), but neuronal damage (*p* < 0.001) and the number of TUNEL-positive cells (*p* < 0.01) were significantly decreased in the LPS + OGD/R + celecoxib group compared with the LPS + OGD/R group.

Flow cytometry assays and western blotting showed the same findings as the CCK8 and LDH leakage rate assays and TUNEL staining. The results of the flow cytometry assay are presented in Figure 5(b). Prenatal LPS exposure and offspring primary neurons exposed to OGD/R significantly increased neuronal apoptosis, but celecoxib significantly decreased neuronal apoptosis. As shown in Figure 5(c), the expression of caspase-3, cleaved caspase-3, and caspase-9 (*p* < 0.05 and *p* < 0.01) was significantly increased in the LPS + N group compared with the N + N group. The expression of caspase-3, cleaved caspase-3, and caspase-9 was significantly increased, but Bcl-2 was significantly decreased in the N + OGD/R group and LPS + OGD/R group compared with the N + N group and the LPS + N group (*p* < 0.05 and *p* < 0.001). The expression of caspase-3, cleaved caspase-3, and caspase-9 was significantly increased in the LPS + OGD/R group compared with the N + OGD/R group (*p* < 0.05, *p* < 0.01, and *p* <0.001). The expression of caspase-3 was significantly increased, but Bcl-2 was significantly decreased in the LPS + N + celecoxib group compared with the LPS + N group (*p* < 0.01 and *p* < 0.001). The expression of caspase-3, cleaved caspase-3, and caspase-9 was significantly decreased, but Bcl-2 was significantly increased (*p* < 0.05, *p* < 0.01, and *p* < 0.001) in the N + OGD/R + celecoxib group compared with the N + OGD/R group. The expression of caspase-3, cleaved caspase-3, and caspase-9 was significantly decreased in the LPS + OGD/R + celecoxib group compared with the LPS + OGD/R group (*p* < 0.05 and *p* < 0.001).

### 3.6. Celecoxib Inhibited Oxidative Stress and Neuroinflammation in OGD/R Offspring Primary Neurons Treated with Prenatal LPS

**Figure 6:**
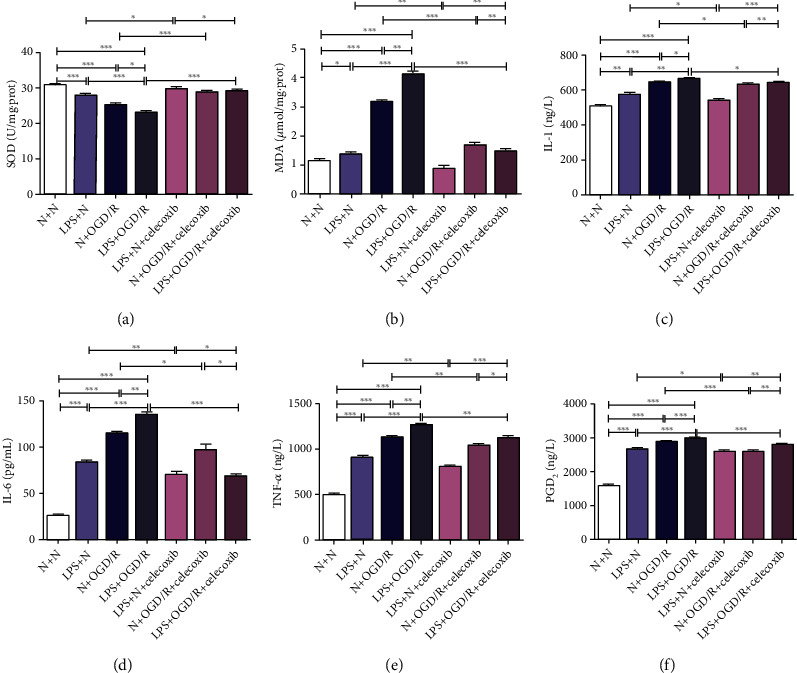
Effect of celecoxib on oxidative stress and inflammation in OGD/R primary offspring neurons treated with prenatal LPS. (a, b) Celecoxib increased SOD activity but decreased MDA content. (c)–(f) Celecoxib decreased levels of IL-1*β*, IL-6, TNF-*α*, and PGD2. Data are expressed as the mean ± SD, *n* = 6. ^∗^*p* < 0.05, ^∗∗^*p* < 0.01, ^∗∗∗^*p* < 0.001.

As shown in Figures 6(a)–6(f), SOD activity (*p* < 0.001) was significantly decreased, but MDA levels (*p* < 0.05) and levels of PGD2, TNF-*α*, IL-1*β*, and IL-6 (*p* < 0.01 and *p* < 0.001) were significantly increased in the LPS + N group compared with the N + N group. SOD activity (*p* < 0.001) was significantly decreased, but MDA levels (*p* < 0.001) and levels of PGD2, TNF-*α*, IL-1*β*, and IL-6 (*p* < 0.01 and *p* < 0.001) were significantly increased in the N + OGD/R group and the LPS + OGD/R group compared with the N + N group and LPS + N group. SOD activity (*p* < 0.05) was significantly decreased, but MDA levels (*p* < 0.01) and levels of PGD2, TNF-*α*, IL-1*β*, and IL-6 (*p* < 0.05, *p* < 0.01, and *p* < 0.001) were significantly increased in the LPS + OGD/R group compared with the N + OGD/R group. SOD activity (*p* < 0.05) was significantly increased, but MDA levels (*p* < 0.01) and levels of PGD2, TNF-*α*, IL-1*β*, and IL-6 (*p* < 0.05 and *p* < 0.01) were significantly decreased in the LPS + N + celecoxib group compared with the LPS + N group. SOD activity (*p* < 0.001) was significantly increased, but MDA levels (*p* < 0.001) and levels of PGD2, TNF-*α*, IL-1*β*, and IL-6 (*p* < 0.05, *p* < 0.01, and *p* < 0.001) were significantly decreased in the N + OGD/R + celecoxib group compared with the N + OGD/R group. SOD activity (*p* < 0.001) was significantly increased, but MDA levels (*p* < 0.001) and levels of PGD2, TNF-*α*, IL-1*β* and IL-6 (*p* < 0.05, *p* < 0.01, and *p* < 0.001) were significantly decreased in the LPS + OGD/R + celecoxib group compared with the LPS + OGD/R group.

### 3.7. Celecoxib Downregulated COX-2/DP_1-2_ Signaling in OGD/R Offspring Primary Neurons Treated with Prenatal LPS

As shown in Figure 5(c), the expression of COX-2, DP_1_, and DP_2_ was significantly increased in the LPS + N group compared with the N + N group (*p* < 0.001). The expression of COX-2, DP_1_, and DP_2_ was significantly increased in the N + OGD/R group and LPS + OGD/R group compared with the N + N group and the LPS + N group (*p* < 0.01 and *p* < 0.001). The expression of COX-2, DP_1_, and DP_2_ was significantly increased in the LPS + OGD/R group compared with the N + OGD/R group (*p* < 0.001). The expression of COX-2, DP_1_, and DP_2_ was significantly decreased in the LPS + N + celecoxib group compared with the LPS + N group (*p* < 0.01). The expression of COX-2, DP_1_, and DP_2_ was significantly decreased in the N + OGD/R + celecoxib group compared with the N + OGD/R group (*p* < 0.01 and *p* < 0.001). The expression of COX-2, DP_1_, and DP_2_ was significantly decreased in the LPS + OGD/R + celecoxib group compared with the LPS + OGD/R group (*p* < 0.01 and *p* < 0.001).

### 3.8. DP_1_ Increased Neuronal Viability and Decreased Neuronal Damage and Neuronal Apoptosis, and DP_2_ Decreased Neuronal Viability and Increased Neuronal Damage and Neuronal Apoptosis in OGD/R Offspring Primary Neurons Treated with Prenatal LPS

**Figure 7:**
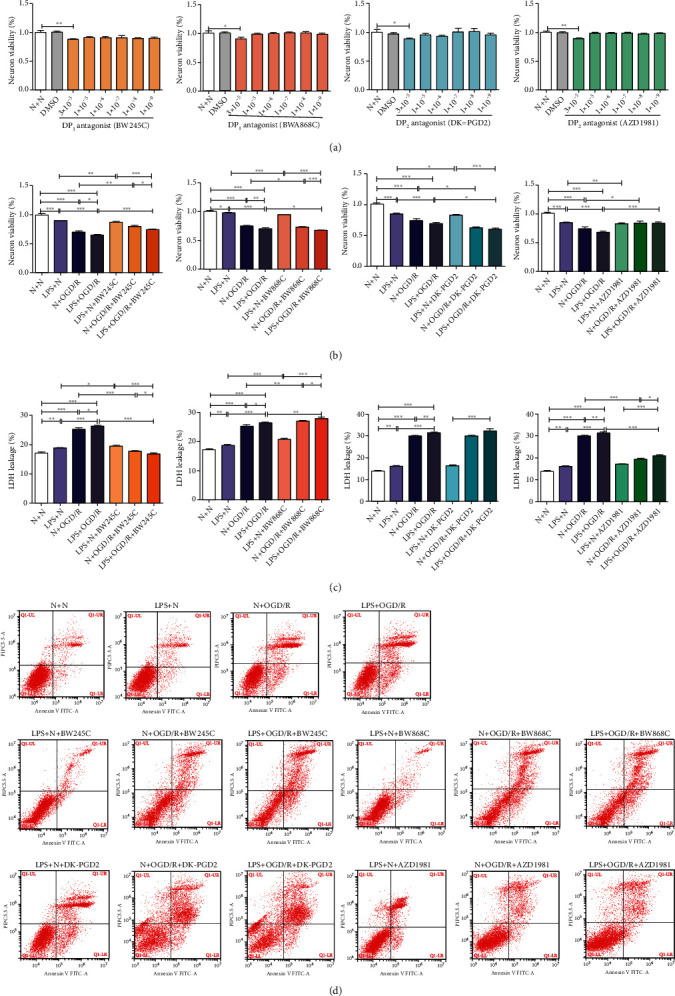
Effect of DP_1_ agonist/antagonist and DP_2_ agonist/antagonist on neuronal viability, neuronal damage and neuronal apoptosis in OGD/R offspring primary neurons treated with prenatal LPS. (a) The concentration of DP_1_ agonist/DP_1_ antagonist/DP_2_ agonist/DP_2_ antagonist showed no toxicity ranging from 1 × 10^−5^ to 1 × 10^−9^ M. (b) DP_1_ agonist and DP_2_ antagonist increased neuronal viability, but DP_1_ antagonist and DP_2_ agonist decreased neuronal viability. (c) DP_1_ antagonist and DP_2_ agonist increased neuronal damage, but DP_1_ agonist and DP_2_ antagonist decreased neuronal damage. (d) DP_1_ antagonist/DP_2_ agonists increased neuronal apoptosis, but DP_1_ agonists/DP_2_ antagonist decreased neuronal apoptosis detected by flow cytometry. Data are expressed as the mean ± SD, *n* = 10. ^∗^*p* < 0.05, ^∗∗^*p* < 0.01, ^∗∗∗^*p* < 0.001.

No toxicity was observed at concentrations ranging from 1 × 10^−5^ to 1 × 10^−9^ M DP_1_ agonist, DP_1_ antagonist, DP_2_ agonist, and DP_2_ antagonist, as shown in Figure 7(a). A concentration of 10^−5^ M for DP_1_ agonist/antagonist and DP_2_ agonist/antagonist was chosen for the follow-up experiments.

As shown in Figures 7(b) and 7(c), the neuronal viability (*p* < 0.01 and *p* < 0.001) was significantly decreased, but neuronal damage (*p* < 0.01) was significantly increased in the LPS + N group compared with the N + N group. The neuronal viability (*p* < 0.001) was significantly decreased, but neuronal damage (*p* < 0.001) was significantly increased in rats in the N + OGD/R group and LPS + OGD/R group compared with that of rats in the N + N group and the LPS + N group. The neuronal viability (*p* < 0.05 and *p* < 0.01) was significantly decreased, but neuronal damage (*p* < 0.05 and *p* < 0.01) was significantly increased in the LPS + OGD/R group compared with the N + OGD/R group. The DP_1_ agonist (BW245C) and DP_2_ antagonist (AZD1981) significantly increased neuronal viability (*p* < 0.001) but significantly decreased neuronal damage (*p* < 0.001). DP_1_ antagonist (BWA868C) and DP_2_ agonist (DK-PGD2) significantly decreased neuronal viability (*p* < 0.05) but significantly increased neuronal damage (*p* < 0.01).

Flow cytometry assays showed that prenatal LPS exposure and offspring primary neurons exposed to OGD/R significantly increased neuronal apoptosis. DP_1_ agonists (BW245C) and DP_2_ antagonist (AZD1981) significantly decreased neuronal apoptosis, while DP_1_ antagonist (BWA868C) and DP_2_ agonists (DK-PGD2) significantly increased neuronal apoptosis (Figure 7(d)).

## 4. Discussion

Epidemiologic and experimental findings implicate maternal infection/inflammation in the etiology of brain injury in preterm newborns [47]. Inflammation is a key factor in the link between infections during pregnancy and neurodevelopmental disorders [48]. Here, our purpose was to assess the effect of maternal inflammation on susceptibility to cerebral I/R injury in offspring and further reveal whether the mechanism is related to the COX-2/PGD2/DP pathway.

It is well known that LPS is effective in inducing an inflammatory response; this concept is usually applied in animal models to induce systemic inflammation [49, 50]. Abundant evidence indicates that maternal LPS exposure promoted increased proinflammatory cytokines in the fetal brain [51, 52]. These neuroinflammatory mediators can activate microglia, leading to neuronal excitation or neuronal loss [53]. Preterm newborns exposed to intrauterine inflammation are at an increased risk of neurodevelopmental disorders [54–57]. Our results showed that neurological deficit scores and cerebral infarction did not differ significantly in offspring rats under maternal LPS exposure but significantly increased the inflammatory response in offspring rats and offspring primary neurons. The precise molecular mechanism underlying brain and behavioral disorders induced by fetal brain inflammation remains poorly understood. However, interestingly, peripheral and central inflammation displayed signs of activation earlier than behavioral and pharmacological impairments related to schizophrenia in models of maternal immunity [58–61], and the change in inflammation may not necessarily be converted to significant behavioral disorders [9].

Oxidative stress can accelerate inflammation directly and indirectly, which further increases oxidative stress [62]. In the elevated inflammation, we observed that maternal LPS exposure aggravated oxidative stress in the hippocampus and cortex of offspring rats and offspring primary neurons. Apoptosis is inextricably linked with excessive inflammatory cascades and oxidative stress [63]. In our study, we observed that in both offspring rats and offspring primary neurons, maternal LPS exposure increased the level of neuronal apoptosis but decreased the expression of antiapoptotic proteins.

The fetal tissues and organs in the sensitive period of development show permanent or programming changes in their structure and function because of the adverse intrauterine environment. These changes might significantly increase offspring susceptibility to a variety of chronic diseases, such as metabolic syndrome, fatty liver, and depressive disorder. The development processes of these diseases are accompanied by inflammatory and immune changes [64, 65]. Early exposure to infection and/or inflammation might induce underlying neuroinflammatory disorders, and these abnormalities could result in progressive disease due to additional exposure to environmental stimuli during postnatal life [66]. This is in good agreement with a “double-hit” model, indicating that the etiologies of diseases might be involved in multiple environments during the different phases of brain development [67–69]. Our research demonstrated that compared to MCAO/R or OGD/R treatment alone, maternal LPS exposure exacerbated neurologic deficits and cerebral infarction volume in MCAO/R offspring rats and aggravated the inflammatory response, oxidative stress, and apoptosis in MCAO/R offspring rats and OGD/R offspring primary neurons. These results indicated that maternal inflammation increased offspring susceptibility to cerebral I/R injury.

COX-2 is a critical enzyme for exacerbating inflammation by catalyzing PGs, and the expression of COX-2, which is closely involved in cerebral I/R injury, was observed in our preliminary experiments [70]. Thus, we observed whether the COX-2 pathway is involved in primary neuronal injury in offspring caused by maternal inflammatory exposure. The results showed that maternal LPS exposure caused derangements of the COX-2 pathway in primary neurons of offspring. Notably, compared to OGD/R treatment alone, maternal LPS exposure exacerbated the disorder of the COX-2 pathway in OGD/R-treated offspring primary neurons. Furthermore, we found that the effect of maternal LPS exposure on offspring was reversed by the COX-2 inhibitor celecoxib.

The findings in the current study indicated that PGD2 had a neuroprotective effect on glutamate-induced neuronal damage [71], and that PGD2 caused several proapoptotic effects in hippocampal neuron injury induced by PPAR*γ* ligands [72]. The COX-2 expression is positively correlated with DP_2_ but negatively correlated with DP_1_ in T2DM rats [73]. DP_1_ was found to have protective effects against permanent focal cerebral ischemia-induced brain injury in mice [74]. DP_2_ mediates Ca^2+^ upregulation and activates protein kinase C (PKC), causing significant activation of inflammatory and immune pathways [75, 76]. We observed that DP_1_ inhibited the OGD/R-induced injury of offspring primary neurons exacerbated by maternal inflammation, including increased neuronal viability and decreased neuronal apoptosis/damage. However, we found that DP_2_ potentiated the OGD/R-induced injury of offspring primary neurons exacerbated by maternal inflammation, including increased neuronal apoptosis/damage and decreased neuronal viability. Overall, our results indicated that maternal inflammation increased offspring susceptibility to cerebral I/R injury via activation of the COX-2/PGD2/DP_2_ pathway.

The clear role of maternal inflammation observed in our study indicated that inhibition of maternal inflammation could exert a neuroprotective effect in offspring. Our study provides further preclinical evidence that maternal inflammation could exert a neuroprotective effect mainly through the COX-2/PGD2/DP_2_ pathway. There is a strong association among maternal infection/inflammation, brain development abnormalities, and brain injuries/susceptibility to brain injuries. Our results clearly show that inhibition of maternal inflammation represents an effective preventative and therapeutic protective strategy in the prenatal environment. Of course, there are several limitations to our study. First, we observed activation of the COX-2/PGD2/DP_2_ pathway post-MCAO/R. However, how maternal inflammation activates the COX-2 pathway is unknown. In subsequent studies, we will focus on whether this effect of maternal inflammation on COX-2 is direct or indirect. Second, glial cells, including microglia and astrocytes, are the major mediators of neuroinflammation [77]. Further research is needed to explore the unique modulatory effects of neuronal-glial cell cocultures and whether our findings could be observed in glial cells. Third, our study focused on PGD2; what about PGE2? Were PGE2 and receptors also further induced by maternal inflammation? Can blocking PGE2 synthesis/signaling have similar effects? In follow-up research, we will study PGD2 and PGE2 interventions simultaneously to further clarify the downstream effects of the maternal inflammation-mediated increase in the COX-2 protein expression.

## 5. Conclusions

**Figure 8:**
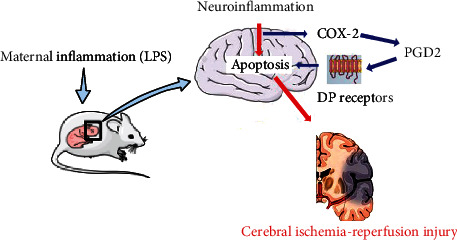
Schematic presentation of the susceptibility to cerebral I/R injury in offspring increased by maternal inflammation. Maternal inflammation exacerbates the inflammatory response, oxidative stress, and neuronal apoptosis via the activation of the COX-2/PGD2/DP_2_ pathway, which in turn increases offspring susceptibility to cerebral I/R injury.

Maternal inflammation exacerbated neuronal apoptosis through the upregulation of proinflammatory cytokines and oxidative stress, subsequently contributing to brain injury in offspring rats and primary neuron injury in offspring. More importantly, maternal inflammation increased susceptibility to cerebral I/R injury both in offspring rats and offspring primary neurons through the above mentioned processes. Moreover, the increased susceptibility to OGD/R in primary neurons of offspring was mediated by the activation of the COX-2/PGD2/DP_2_ pathway. Inhibition of COX-2 and DP_2_ and activation of DP_1_ decreased, while inhibition of DP_1_ and activation of DP_2_ increased the susceptibility to OGD/R-induced primary neuron injury in offspring. Schematic presentation of the susceptibility of the offspring to cerebral I/R injury increased with maternal inflammation (Figure 8). Maternal inflammatory exposure caused an inflammatory response, oxidative stress, and neuronal apoptosis via the activation of the COX-2/PGD2/DP_2_ pathway, ultimately increasing offspring susceptibility to cerebral I/R injury.

## Data Availability

All data generated or analyzed during this study are included in this published article (and Supplementary Information).

## Abbreviations

Cerebral ischemia–reperfusion injury: Cerebral I/R injury
COX-2: Cyclooxygenase-2
DP_1_: D-type prostanoid receptor 1
DP_2_: D-type prostanoid receptor 2
HE: Hematoxylin and eosin
IL-1*β*: Interleukin 1*β*
IL-6: Interleukin 6
MCAO/R: Middle cerebral artery occlusion and reperfusion
MDA: Malondialdehyde
OGD/R: Oxygen-glucose deprivation and reoxygenation
PGD2: Prostaglandin D2
SOD: Superoxide dismutase
TNF-*α*: Tumor necrosis factor-*α*
TTC: Triphenyltetrazolium chloride.

## Supplementary Materials

Supplementary Materials Figure S1: morphological characteristics and identification of primary neurons. (a) Primary neuron morphological characteristics were observed after 1, 3, 5, and 7 days of culture. Scale bar = 100 *μ*m. (b) Neurons were identified by the neuronal marker NeuN after 7 days of culture. The purity of the neurons was >95%. Scale bar = 100 *μ*m and 50 *μ*m. Figure S2: determine the most efficient OGD/R treatment time. A neuronal viability was analyzed by a CCK-8 assay. (b) Neuronal damage was analyzed by an LDH leakage rate assay. Primary neurons subjected to 2 h OGD treatment and 24 h reoxygenation were the most efficient treatment time and were chosen for the follow-up experiments. Data are expressed as the mean ± SD, *n* = 10. ^∗∗∗^*p* < 0.001.
