# Supplementary material for: Maternal Inflammation Exaggerates Offspring Susceptibility to Cerebral Ischemia–Reperfusion Injury via the COX-2/PGD2/DP2 Pathway Activation
Source: Oxid Med Cell Longev. 2022 Apr 9;2022:1571705. doi: 10.1155/2022/1571705 (PMC9013311; doi:10.1155/2022/1571705)
Supplement: Supplementary Materials — Figure S1: morphological characteristics and identification of primary neurons. (a) Primary neuron morphological characteristics were observed after 1, 3, 5, and 7 days of culture. Scale bar = 100 μm. (b) Neurons were identified by the neuronal marker NeuN after 7 days of culture. The purity of the neurons was >95%. Scale bar = 100 μm and 50 μm. Figure S2: determine the most efficient OGD/R treatment time. A neuronal viability was analyzed by a CCK-8 assay. (b) Neuronal damage was analyzed by an LDH leakage rate assay. Primary neurons subjected to 2 h OGD treatment and 24 h reoxygenation were the most efficient treatment time and were chosen for the follow-up experiments. Data are expressed as the mean ± SD, n = 10. ∗∗∗p < 0.001. [file 1571705.f1.docx]

**Supplementary Materials (*Oxidative Medicine and Cellular Longevity*)**

**Maternal Inflammation Exaggerates Offspring Susceptibility to Cerebral Ischemia-Reperfusion Injury via the COX-2/PGD2/DP_2_ Pathway Activation**

Yuke Li,^1,†^ Wen Luo,^2,†^ Jiahua Zhang,^1^ Ying Luo,^1^ Wenli Han,^3^ Hong Wang,^1^ Hui Xia,^1^ Zhihao Chen,^1^ Yang Yang,^1^ Qi Chen,^4^ Huan Li,^1^ Lu Yang,^1^ Congli Hu,^1^ Haifeng Huang,^1^ Zhe Peng,^1^ Xiaodan Tan,^1^ Miaomiao Li,^1^ and Junqing Yang^1,*^

^1^ College of Pharmacy, Chongqing Medical University, Chongqing Key Laboratory of Biochemistry and Molecular Pharmacology, Chongqing 400016, China

^2^ Department of Clinical Pharmacy, The Third Hospital of Mianyang/Sichuan Mental Health Center, Mianyang, 621000, China

^3^ Laboratory animal center, Chongqing Medical University, Chongqing 400016, China

^4^ Pharmacy Department of Guizhou Provincial People^’^s Hospital, Guiyang 550000, China

* Correspondence should be addressed to Junqing Yang; cqyangjq@cqmu.edu.cn

^†^Yuke Li and Wen Luo contributed equally to this work.

**
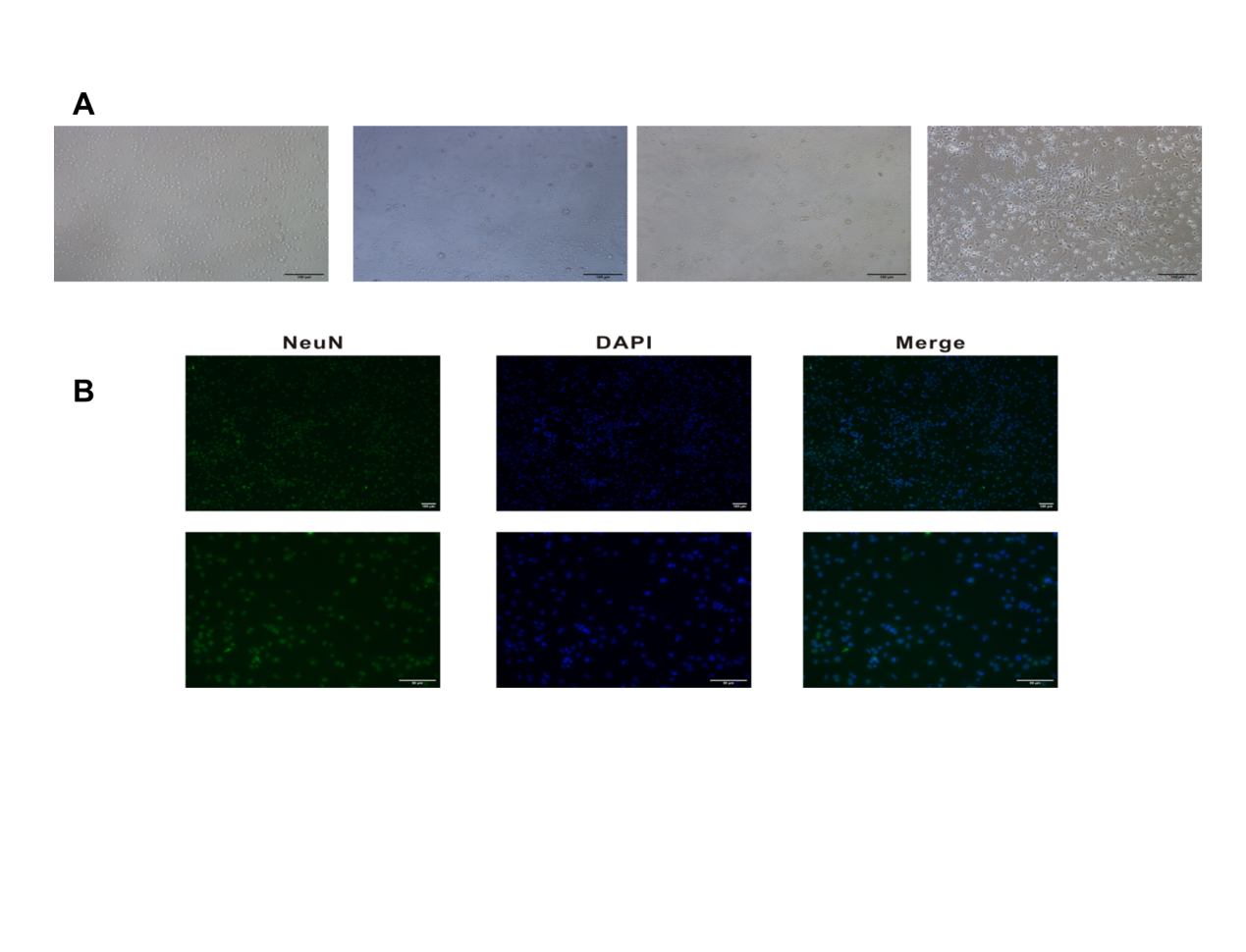
**

Figure S1: The morphological characteristics and identification of primary neurons. A Primary neuron morphological characteristic was observed after 1, 3, 5, and 7 days of culture, respectively. Scale bar = 100µm. B Neurons were identified by the neuronal marker NeuN after 7 days of culture. The purity of the neurons was >95%. Scale bar = 100µm and 50µm.

**
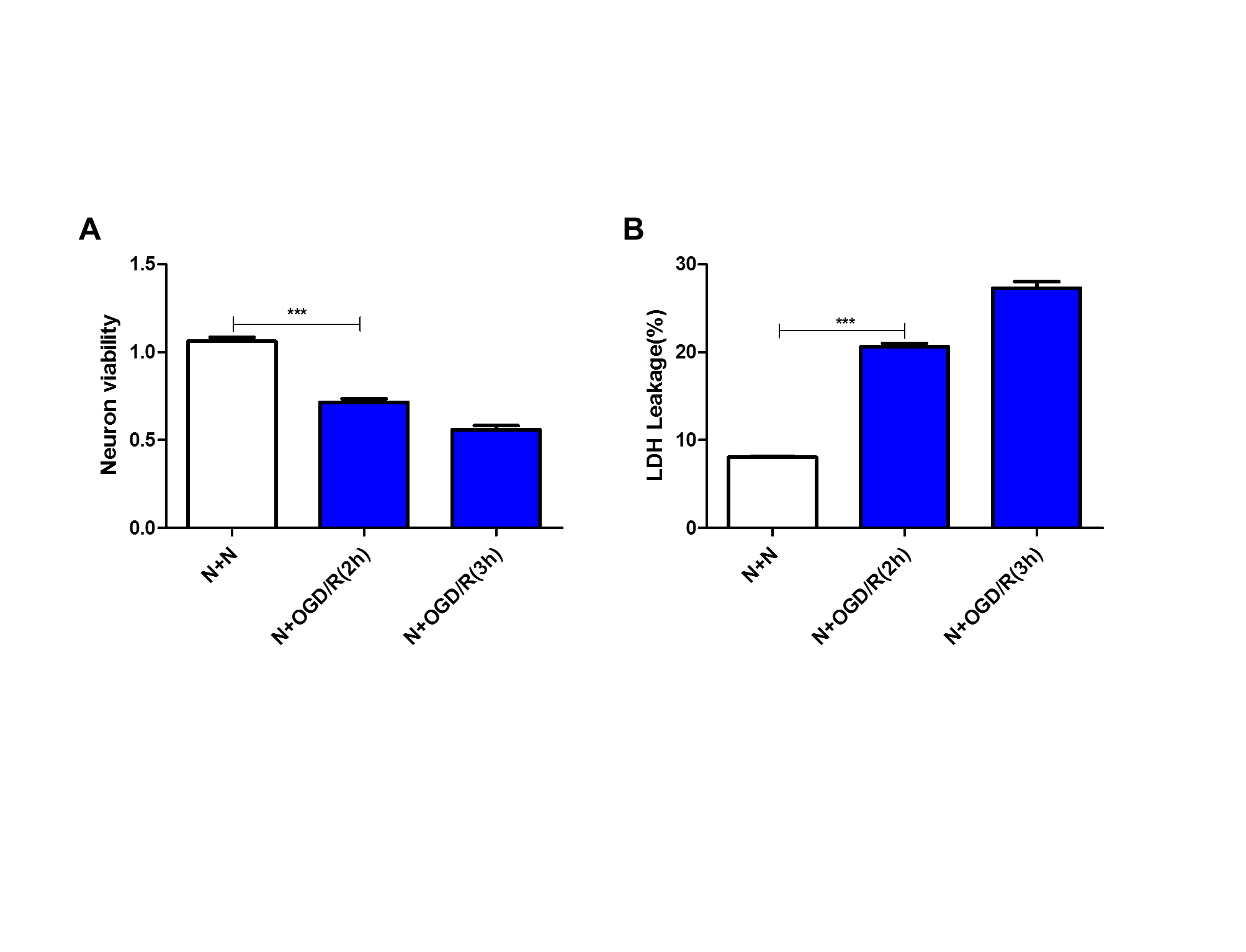
**

Figure S2: Determine the most efficient OGD/R treatment time. A Neuronal viability was analyzed by a CCK-8 assay. B Neuronal damage was analyzed by an LDH leakage rate assay. Primary neurons subjected to 2h OGD treatment and 24 h reoxygenation were the most efficient treatment time and were chosen for the follow-up experiments. Data are expressed as the mean ± SD, n=10. *** *p* < 0.001
